# Distributed and Lumped Parameter Models for the Characterization of High Throughput Bioreactors

**DOI:** 10.1371/journal.pone.0162774

**Published:** 2016-09-26

**Authors:** Laura Iannetti, Giovanna D’Urso, Gioacchino Conoscenti, Elena Cutrì, Rocky S. Tuan, Manuela T. Raimondi, Riccardo Gottardi, Paolo Zunino

**Affiliations:** 1 Department of Chemistry, Materials and Chemical Engineering “Giulio Natta”, Politecnico di Milano, Milan, Italy; 2 Department of Chemical, Industrial, Computer, Mechanical Engineering, Università degli Studi di Palermo, Palermo, Italy; 3 Fondazione Ri.MED, Palermo, Palermo, Italy; 4 Department of Orthopaedic Surgery, University of Pittsburgh, Pittsburgh, Pennsylvania, United States of America; 5 McGowan Institute for Regenerative Medicine, University of Pittsburgh, Pittsburgh, Pennsylvania, United States of America; 6 MOX, Department of Mathematics, Politecnico di Milano, Milan, Italy; 7 Department of Mechanical Engineering and Materials Science, University of Pittsburgh, Pittsburgh, Pennsylvania, United States of America; Michigan Technological University, UNITED STATES

## Abstract

Next generation bioreactors are being developed to generate multiple human cell-based tissue analogs within the same fluidic system, to better recapitulate the complexity and interconnection of human physiology [1, 2]. The effective development of these devices requires a solid understanding of their interconnected fluidics, to predict the transport of nutrients and waste through the constructs and improve the design accordingly. In this work, we focus on a specific model of bioreactor, with multiple input/outputs, aimed at generating osteochondral constructs, i.e., a biphasic construct in which one side is cartilaginous in nature, while the other is osseous. We next develop a general computational approach to model the microfluidics of a multi-chamber, interconnected system that may be applied to human-on-chip devices. This objective requires overcoming several challenges at the level of computational modeling. The main one consists of addressing the multi-physics nature of the problem that combines free flow in channels with hindered flow in porous media. Fluid dynamics is also coupled with advection-diffusion-reaction equations that model the transport of biomolecules throughout the system and their interaction with living tissues and C constructs. Ultimately, we aim at providing a predictive approach useful for the general organ-on-chip community. To this end, we have developed a lumped parameter approach that allows us to analyze the behavior of multi-unit bioreactor systems with modest computational effort, provided that the behavior of a single unit can be fully characterized.

## 1 Introduction

A number of *in vitro* approaches have been used over time for high throughput drug screening or toxicology testing. However, most currently available systems are only partial approximations of human biology and their predictive capacity is consequently limited. In fact, such systems are either based on human cell cultures, not capturing the complexity of cell behavior in a three dimensional (3D) environment, or they are based on animal tissues fragments, 3D in nature but only partially biosimilar to human tissues and unable to account for interactions with other organs. To overcome these limitations, next generation bioreactors are being developed to generate multiple human cell-based tissue analogs within the same fluidic system to better recapitulate the complexity and interconnection of human physiology. These efforts aim at creating multi-tissue organ systems (cardiovascular, gastro-intestinal, musculoskeletal, etc.) that ultimately can be joined in an interconnected human-on-chip device capable of providing a veritable representation of the body complex response to diseases and potential drug treatments [3–5].

**Fig 1:**
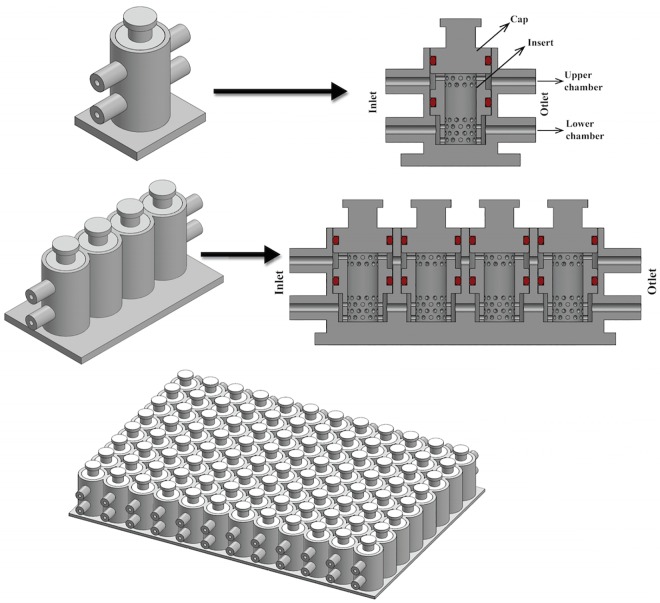
Different bioreactor configurations. 1 cell (top left), 1-unit in cross section (top right), 4-units (bottom left) and 96-units. (bottom right).

The effective development of these devices requires a solid understanding of their interconnected fluidics, to predict the transport of nutrients and waste through the constructs and improve the design accordingly. In this work, we have focused on a specific bioreactor with multiple input/output aimed at generating osteochondral constructs, i.e., a biphasic constructs in which one side is cartilaginous in nature, while the other is osseous. This bioreactor [1, 6, 7] represented in Fig 1 has been chosen since it comprises both a dual chamber system to host a single biphasic tissue construct with distinct fluidics (Fig 1, top), and a set of interconnected chambers with common fluidics (Fig 1, bottom). Starting from this specific bioreactor, we have developed a general approach to model the microfluidics of a multi-chamber, interconnected system that may be applied to human-on-chip devices.

The microphysiological osteochondral bioreactor analyzed in this work is aimed at the study of osteoarthritis (OA), a major pathology of articular joints, affecting over 33% of the population over the age of 65 [8]. The hallmark of this disease that affects all tissues in the joint, is the progressive degeneration of cartilage which begins well before clinical symptoms manifest, ultimately requiring joint replacement surgery [9]. The high incidence of this painful and disabling pathology begs for the understanding of the causes and mechanisms of its development, in order to identify reparative drug therapies to arrest or even regenerate the damaged tissues and ultimately avoid surgery. A novel strategy in this respect adopts a tissue engineering approach and the use of bioreactors [1, 7] to generate a high number of identical *in vitro* constructs that can replicate the pathogenesis of joint diseases for the identification of therapeutic targets and for drug screening [1, 10–12]. Critical in this respect is the development of a representative model of the interactions between cartilage and other joint tissues and, in particular, with the subchondral bone. In fact, there is growing evidence of the exchange of nutrients, cytokines, and hormones *in vivo* between bone and cartilage. The osteochondral (OC) unit is then conceived as the main target of OA, to reflect the dynamic cartilage/bone interplay in both health and disease [1, 13–18]. The medium to high throughput system studied in this work, which we call high-throughput bioreactor (HTB) hereon, is the first of its kind. It hosts in a single chamber a biphasic construct, with separate fluidics for its cartilaginous and osseous components, effectively creating a dual-chamber setup (Fig 1) [6, 7]. In this way, cartilage and bone will be in contact and able to signal to each other, while each is exposed to its ideal culture medium. Furthermore, the HTB allows the generation and culture of a high number of identical OC constructs similar in dimensions to native tissue biopsies [1, 6, 7]. It must be noted that the physiological functions of the examined tissue are primarily load bearing and force transduction, which imply a key role for the extracellular matrix (ECM), also an essential player in the regulation of cell differentiation, physiology and response to insults [1, 19, 20]. Consequently, a bioreactor that accommodates a significant ECM tissue component to recapitulate at least some of the physiological aspects of the osteochondral complex requires a relatively larger volume, in the order of millimeters rather than the hundreds of micrometers more common in microfluidic systems. To generate a construct that mimics tissue physiology, the bioreactor chamber is filled with a cell-laden porous polymeric scaffold. Hence, the larger size and the presence of porous scaffold within the insert makes nutrient perfusion within the device a potential challenge, since to avoid cellular hypoxia and to obtain adequate tissue development, nutrients must travel a longer path to reach the inner regions within the bioreactor. In this context, we use computational fluid dynamics to assess the hydrodynamic properties of the system. Previous works [4, 21–23] evaluated the fluid mixing and transport of nutrients between chambers in the same unit of a forced perfusion setup, but to our knowledge there are no similar studies about the interaction of fluid and porous constructs in a design with more effective fluidics as the one in Fig 1.

Furthermore, to achieve a high-throughput drug screening system, single bioreactor dual-chambers (bioreactor unit) have been connected and combined in a multi-unit system, organized in sequential and parallel rows (Fig 1). In the 96 wells design presented in Fig 1, individual units are connected only in series, 8 at a time as this design is best suited for drug or toxicological screening; to asses for instance a dose response, each array of 8 units can be subjected to a different concentration of the compound under examination. “In parallel” connection, although possible, has not been envisioned. The constructs in each row are meant as replicates for multiple endpoint testing (e.g., histology, PCR, etc.). A further challenge is then to guarantee that the tissue constructs in the downstream chambers receive the appropriate amount of nutrients from the fluid that has perfused the units upstream. In other words, not only a dual-chamber bioreactor, but also a multi-unit array shall be analyzed.

The specific objective of this work is to develop a methodology to characterize the flow and transport in a HTB by means of a computational modeling approach, combining distributed and lumped parameter models. In particular, we have assessed the degree of perfusion and mixing of nutrients in each region of the device, evaluating the effect of different scaffold types. The computational model was then used to compare two different engineered constructs, a hydrogel (methacrylated gelatin, GelMA [6, 24]) and a porous polymeric scaffold (poly-L-lactate, PLLA)[25]. The first one features very small pore size and is solute permeable, the second one shows larger pore size and is impenetrable to fluid and nutrients.

Performing such simulations requires overcoming several challenges at the level of computational modeling. The main one consists of addressing the multi-physics nature of the problem that combines free flow in channels with hindered flow in porous media. Fluid dynamics is then coupled with advection-diffusion-reaction equations that model the transport of biomolecules throughout the system and their interaction with living tissue. Besides these modeling challenges, the complex configuration of the bioreactor poses significant difficulties in building the CAD model and discretizing its parts with a computational mesh suitable for the application of a numerical scheme. These issues can be solved using an in-house-made software that incorporates state-of-the-art efficient algorithms for the approximation of partial differential equations. Although this approach is viable, it entails significant costs in terms of man-hours for the implementation and validation of the new software. For this reason, we have adopted here a commercial platform, ANSYS (ANSYS Inc., Canonsburg, PA), which features advanced multi-physics simulation capabilities. Another challenging aspect of this work is then to stretch the limits of the ANSYS platform to address the complex problem at hand. Ultimately, our aim is to provide a predictive approach useful for the general organ-on-chip community. To this end, we have developed a lumped parameter approach that allows us to analyze the behavior of multi-unit bioreactor systems with a modest computational effort, provided that the behavior of a single unit could be fully characterized. If the linearity conditions are satisfied, this computational methodology is independent from the specific osteochondral nature of the biological system being studied. Our approach simply describes a network of interconnected multi-chamber units. Consequently, we believe that our approach can be directly applied to predict the flow and transport of a generic human-on-chip setup, even those comprising multiple physiological systems (e.g., a liver model connected to a kidney model, connected to a bone model, etc.) with single or multi-chamber units.

## 2 Models and Methods

Exploiting the commercial platform ANSYS (ANSYS Inc., Canonsburg, PA), we have developed a CAD model of the bioreactor and we have used it to simulate flow and transport phenomena in the system. The steps to achieve a realistic simulation of the fluid and transport within the bioreactor are detailed below.

### 2.1 CAD model

The 3D CAD model of the bioreactor was created using ANSYS ICEM CFD v.15.0 (ANSYS Inc.) CAD modeler. We have considered a row of 4-units connected in series (see Fig 1). Each unit has the same configuration, specifically designed to grow a construct that combines cartilage and bone, and comprises the following parts: two inlets and two outlets consisting of cylindrical channels, to guarantee the circulation of fluid from the upstream units to the downstream ones. Each inlet/outlet channel is characterized by a length (L) of 5.3 mm and an inner diameter (d) of 1 mm. The perforated cylindrical insert that holds the scaffold in place is 8.5 mm high and 3.75 mm wide. Each bioreactor chamber is sealed by an upper cap and by two O-rings (see Fig 1). Forthcoming extensions of this study will consider rows of 8 bioreactor units. By aligning 12 parallel lines of these rows, one obtains a plate of 96-units, which is a realistic prototype of high-throughput bioreactor for drug screening.

### 2.2 Flow

**Fig 2:**
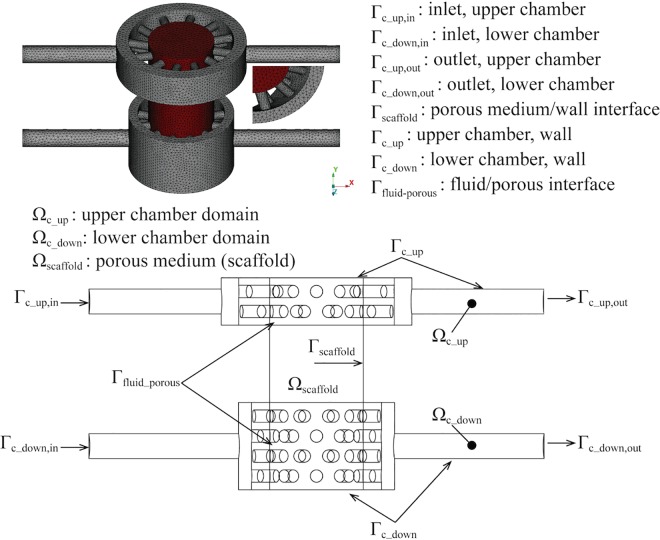
Representation of the bioreactor. Free fluid regions are visualized in grey, the porous medium is red (top). For the localization of boundary surfaces, Ω and Г indicate volume and surface, respectively (bottom).

The bioreactor features the combination of free flow for the inlets, outlets, and the outer chambers with porous media flow for the inner culture chamber (insert). In each region, we assume that the flow is incompressible. For momentum balance, our approach employs a general equation that encompasses the nature of both types of flow, and we will switch between them by suitably tuning the problem parameters in each region. This equation has the structure of Brinkman equation for flow in porous media, because it combines viscous terms, such as in Stokes, with friction terms, such as in Darcy. To model free flow, a convective term, which plays a significant role in case of high Reynolds regimes, was added. Static conditions are also assumed. Then, the momentum balance equation reads as follows:

$$\nabla ∙(\rho \underline{U}\times \underline{U})-\nabla ∙\left(\mu \;(\nabla \underline{U}{+(\nabla \underline{U})}^{T})\right)=-\frac{\mu }{K_{perm}}\underline{U}-\nabla p\;\forall x\in {Ω}_{\text{c}\_\text{up}}\cup {Ω}_{c_{\text{down}}}\cup {Ω}_{\text{scaffold}}$$ (1)

where $\underline{U}$ denotes the velocity vector field ($\underline{{\underline{U}}_{f}}$ and ${\underline{U}}_{s}$ denote the restriction of the velocity field to the free fluid and porous medium, respectively), *p* the hydrostatic pressure, *ρ* e *μ* are the fluid viscosity and density respectively, and *K*_*perm*_ the hydraulic conductivity of the porous medium (for the free flow regions we set *K*_*perm*_ → ∞). For the partition of the bioreactor into sub-regions, we refer to Fig 2. We assume that the culture medium that perfuses the bioreactor is comparable to water (*ρ* = 999,97 *kg*/*m*^3^ *μ* = 0,001 *Pa s*) since the dissolved nutrients and other chemical species are relatively dilute.

For the definition of boundary conditions, we partition the bioreactor surface as illustrated in Fig 2. At the bioreactor inlet, (*Γ*_c_up,in_ *e Γ*_c_down,in_), a given flow rate is applied through the enforcement of a flat velocity profile on the inflow sections; a *no-slip* condition is adopted on the surfaces that separate the free fluid and the porous medium from the bioreactor walls ($\Gamma_{c\_up},\;\Gamma_{c_{down}}\;e\;\Gamma_{scaffold}$), which have been assumed to be rigid walls. At the outlet, (*Γ*_c_up,out_ *e Γ*_c_down,out_) we have set a uniform normal stress field equal to the atmospheric pressure, namely **σ**_*f*_ ∙ ***n*** = **0**, where $\sigma_{f}=\nabla ∙\left(\mu \;(\nabla \underline{U}{+(\nabla \underline{U})}^{T})\right)-\nabla p$ is the Cauchy stress in the fluid. Given the previous modeling choices, the flow problem becomes

$$\begin{array}{cc} \nabla ∙\underline{U}=0 & \forall x\in {Ω}_{c\_\text{up}}\cup {Ω}_{c\_\text{down}}\cup {Ω}_{\text{scaffold}}\\ \nabla ∙\left(\rho \underline{U}\times \underline{U}\right)-\nabla ∙\left(\mu \;\left(\nabla \underline{U}+{\left(\nabla \underline{U}\right)}^{T}\right)\right)=-\frac{\mu }{K_{perm}}\underline{U}-\nabla p & \forall x\in {Ω}_{\text{c}\_\text{up}}\cup {Ω}_{c\_\text{down}}\cup {Ω}_{\text{scaffold}}\\ Q\left(r\right)=\bar{Q} & \forall x\in \Gamma_{\text{c}\_\text{up},\text{in}}\cup \Gamma_{\text{c}\_\text{down},\text{in}}\\ p=0 & \forall x\in \Gamma_{\text{c}\_\text{up},\text{out}}\cup \Gamma_{\text{c}\_\text{down},\text{out}}\\ \underline{U}=0 & \forall x\in \Gamma_{\text{c}\_\text{up}}\cup \Gamma_{\text{c}\_\text{down}}\cup \Gamma_{\text{scaffold}}\\ {\underline{U}}_{f}={\underline{U}}_{s} & \forall x\in \Gamma_{\text{fluid}-\text{porous}}\\ \sigma_{f}∙n=\sigma_{s}∙n & \forall x\in \Gamma_{\text{fluid}-\text{porous}} \end{array}$$ (2)

### 2.3 Mass transport

An important part of this study consists of modeling the transport of bio-molecules dissolved in the culture media that perfuse the bioreactor. In particular, we focus on oxygen, fundamental to guarantee cell survival. However, the model is general and has been used to describe the transport of glucose and proteins, as it will be reported in forthcoming works.

Since all solutes are diluted, they are modeled as passively transported by the culture media. Their governing equations have been formulated in terms of volumetric concentrations measured in [mg/ml]. The symbol C denotes the solute concentration, D the diffusion coefficient for the specific biomolecule and the subscripts *f* and *s* indicate the fluid and the porous medium (scaffold), respectively. Therefore the equation describing the biomolecules’ transport in the fluid phase is:

$$\nabla ∙(-D_{f}\nabla C_{f}+\underline{U}C_{f})=0\;\forall \;x\in {Ω}_{\text{c}\_\text{up}}\cup {Ω}_{c\_\text{down}}$$ (3)

For the porous medium, namely the scaffold region, we assume that fluid and solid phases coexist. We denote with *C*_*s*,*s*_ and *C*_*s*,*f*_ the volumetric concentration of biomolecules in the solid and in the fluid phase of the scaffold, respectively. Denoting with *γ* the porosity of the scaffold (complement to unity of the solid phase, i.e. for the free flow regions we set *γ* = 1), the volumetric concentration of biomolecules in the porous medium is given by the following weighted average *C*_*s*_ = *γC*_*s*,*f*_ + (1 − *γ*)*C*_*s*,*s*_. Then, following the theory of mixtures, the governing equations for biomolecules concentration in the porous medium read as follows:

$$\begin{array}{c} \nabla ∙(-D_{s,s}\nabla (1-\gamma )C_{s,s}+(1-\gamma )\underline{U}C_{s,s})+(1-\gamma )S+\tau IAD(C_{s,s}-C_{s,f})=0;\\ \nabla ∙(-D_{s,f}\nabla \gamma C_{s,f}+\underline{U}{\gamma C}_{s,f})+\gamma S+\tau IAD(C_{s,f}-C_{s,s})=0\;\forall \;x\in {Ω}_{\text{scaffold}}. \end{array}$$ (4)

This model assumes that both the fluid and the solid phases in the porous medium are permeable to biomolecules. The mass transfer coefficient from the fluid to the solid phase in the porous medium is *τ*, while *IAD* is the interface area density of the surface separating the two phases. As a result, the term *τIAD*(*C*_*s*,*s*_ − *C*_*s*,*f*_) represents the flux exchanged between the two phases of the porous medium. The symbol *S* denotes the source term representing the consumption of nutrients by living cells disseminated into the scaffold. For this reason, it is usually a function (linear or nonlinear) of the nutrient concentration. We will discuss the constitutive models for the parameters *S*,*τ*,*IAD* in the next section.

At the inlet boundaries (*Γ*_c_up,in_ *e Γ*_c_down,in_) a known concentration has been imposed, using independent values on each inlet section. A homogeneous *Neumann* condition ∇*C*_*f*_ ∙ ***n*** = 0 has been adopted on the bioreactor wall and outlets ($\Gamma_{c\_up},\;\Gamma_{c_{down}},\;\Gamma_{c\_up,out}\;e\;\Gamma_{c\_down,out}$). In fact, the wall is considered impermeable to nourishments and their flux in the direction normal to the outlets is assumed equal to zero. Moreover, conservation of concentrations *C*_*f*_ = *C*_*s*_ and of biomolecules flux −*D*_*f*_∇ ∙ *C*_*f*_***n*** = −*D*_*s*_∇ ∙ *C*_*s*_***n*** have been applied at the interface between fluid and porous media (*Γ*_fluid–porous_). As a result, the concentration of oxygen is determined by the following problem:

$$\begin{array}{cc} \nabla ∙\left(-D_{s,f}\nabla \gamma C_{s,f}+\underline{U}\gamma C_{s,f}\right)+\gamma S+\tau IAD\left(C_{s,f}-C_{s,s}\right)=0 & \forall x\in {Ω}_{\mathrm{scaffold}}\\ \nabla ∙\left(-D_{s,f}\nabla \gamma C_{s,f}+\underline{U}\gamma C_{s,f}\right)+\gamma S+\tau IAD\left(C_{s,f}-C_{s,s}\right)=0 & \forall x\in {Ω}_{\text{scaffold}}\\ \nabla ∙\left(-D_{f}\nabla C_{f}+\underline{U}C_{f}\right)=0 & \forall x\in {Ω}_{c\_up}\cup {Ω}_{\text{c}\_\text{down}}\\ C_{f=}{\bar{C}}_{1} & \forall x\in \Gamma_{c\_\mathrm{up},\mathrm{in}}\\ C_{f=}{\bar{C}}_{2} & \forall x\in \Gamma_{c\_\mathrm{down},\mathrm{in}}\\ \nabla C_{f}∙n=0 & \forall x\in \Gamma_{c\_\mathrm{up},\mathrm{out}}\cup \Gamma_{c\_\mathrm{down},\mathrm{out}}\\ \nabla C_{f}∙n=0 & \forall x\in \Gamma_{c\_\mathrm{up}}\cup \Gamma_{c\_\mathrm{down}}\\ C_{f}=\gamma C_{s,f}+\left(1-\gamma \right)C_{s,s} & \forall x\in \Gamma_{\mathrm{fluid}-\mathrm{porous}}\\ -\gamma D_{f}\nabla C_{f}∙n=-D_{s}\nabla \left(\gamma C_{s,f}\right)∙n & \forall x\in \Gamma_{\mathrm{fluid}-\mathrm{porous}}\\ -\left(1-\gamma \right)D_{f}\nabla C_{f}∙n=-D_{s}\nabla \left(\left(1-\gamma \right)C_{s,s}\right)∙n & \forall x\in \Gamma_{\mathrm{fluid}-\mathrm{porous}} \end{array}$$ (5)

### 2.4 Model parameters and constitutive laws

#### 2.4.1 Model parameters for the flow model

First, the characteristic Reynolds number of the flow in the bioreactor was determined from the following definition,

$$Re=\frac{vD\rho }{\mu }=\frac{4}{\pi D}\frac{\rho v\pi D^{2}}{4\mu }=\frac{4}{\pi D}\frac{\rho \bar{Q}}{\mu }$$ (6)

where *D* is the inlet diameter of 1mm, *ρ* = 999,97 *Kg*/*m*^3^ and *μ* = 0,001 *Pa* ∙ *s* are the fluid density and dynamic viscosity, respectively, $\bar{Q}$ is the inlet flow rate into each chamber, equal to 1 ml/day. A *Re* ≪ 0,01, was found thus confirming that the assumption of laminar flow is accurately verified. As a consequence, the inertial (and nonlinear) term in the momentum equation, namely $\rho \underline{U}\times \underline{U}$, can be neglected and the flow model turns out to be a set of linear equations. This will be the key property for the later derivation of a surrogate of the flow model, which is only based on algebraic equations consequently featuring a negligible computational cost.

Another parameter, essential to determining the flow in the porous medium is the (intrinsic) permeability *K*_*perm*_ that is determined by the microscopic structure of the scaffold, quantified by the porosity (*γ*), the tortuosity, etc. In the case of materials featuring an anisotropic structure, permeability is a tensor quantity. Here, since the scaffolds under consideration are isotropic, it becomes a scalar parameter. In what follows, we will consider two types of scaffolds, one made out of methacrylated gelatin (GelMA) and the other consisting of a poly-L-lactate (PLLA) foam. The porosity and permeability of the latter have been estimated via Boyle’s pycnometer and scanning electron microscopy (SEM) analysis. Data for GelMA are scarce in literature. However, for tissue engineering it is used as a surrogate material to mimic the extracellular matrix of cartilage; hence, we initialized the model for the bioreactor configuration using data that have been previously measured for native cartilage [26]. In both cases, the values for porosity and permeability are reported in Table 1.

**Table 1:** Porosity and permeability values used for GelMA and PLLA scaffolds.

	GelMA scaffold	PLLA scaffold
Porosity	0.8	0.93
Permeability [m^2]	1 e-16^[^^20^^]^	3.23384e-09

#### 2.4.2 Model Parameters and constitutive laws for mass transport

Inlet concentrations for oxygen are 3.15 e-3 [mg/ml] and 7.2 e-3 [mg/ml] for the upper and lower chamber, respectively. We observe that the oxygen supply of the upper chamber falls within the range of hypoxic conditions, compatible with the biological need of the chondral tissue, while the lower chamber, where bone is developed, is kept under normoxic conditions. These different environments are aimed at supporting stem cell differentiation into a chondral and osseous phenotype, respectively [27]. The diffusion coefficient was obtained from previously published studies [28].

**Fig 3:**
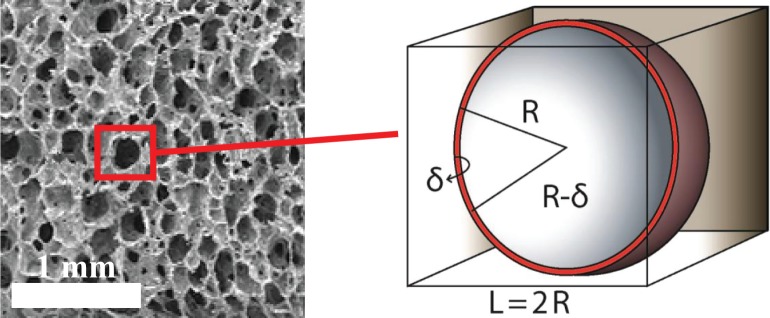
Representative SEM micrograph of the PLLA scaffold and microscopic model of the scaffold pores for the quantification of the exchange between fluid and solid constituents of the porous matrix.

For the exchange of biomolecules between fluid and solid phases within the scaffold, the coefficients *τ*,*IAD* must be calculated. To this purpose, we model the porous medium as a periodic structure whose unit can be idealized as a cube containing a hollow sphere, namely the pore, as illustrated in Fig 3.

Although this configuration is incompatible with the flow through the pore, as it is completely closed, it is adequate for modeling mass transfer between the solid and the fluid phases of the porous medium. According to this model, we estimate the value of the interface area density (*IAD*), which only depends on the configuration of the unit. Let *S*_*e_s*_ = 4*πR*^2^ and *S*_*i_s*_ = 4*π*(*R*−*δ*)^2^ be the external and internal pore surface, respectively, and let *V*_*c*_ be the total volume of the unit. Then the interface area density is defined as:

$$IAD=\frac{S_{e\_s}+S_{i\_s}}{V_{c}}$$ (7)

To estimate the mass transfer coefficient, we assume that at the pore scale mass transfer is dominated by diffusion in the solid phase. As a consequence, the Sherwood number magnitude turns out to be in the range of unity. Exploiting this assumption, we have

$$1=Sh=\frac{\tau d}{D_{s,s}}$$ (8)

where *τ* is the mass transfer coefficient and *d* is the pore diameter. As a result, we obtain,

$$\tau =\frac{D_{s,s}}{d}$$ (9)

We observe that GelMA and PLLA have different behaviors with respect to mass transfer and interface area density. GelMA scaffold has homogeneous properties, namely the pore radius is uniform everywhere and equal to R = 9.77205 e-6[m] with a thickness δ = 10%R. The GelMA matrix is permeable to solutes, as shown by the positive diffusion coefficients *D*_*s*,*s*_ reported in Table 2. The PLLA scaffold is substantially different because it is impermeable to solutes. As a result, the mass transfer coefficient is necessarily null. Since the exchange between solid and fluid phases in the porous medium is modeled by terms *τIAD*(*C*_*s*,*s*_−*C*_*s*,*f*_), we notice that the interface area density does not affect the model.

**Table 2:** Oxygen parameters adopted for GelMA and PLLA scaffolds.

	GelMA	PLLA
*D*_*f*_ = *D*_*s*,*f*_	2.1 x10^-9^ [m^2^/s]	2.1 x10^-9^ [m^2^/s]
*D*_*s*,*s*_	4.5 x10^-10^ [m^2^/s]	0
*τ*	0.230248 e-5^[^^21^^]^ [m s^-1]	0
IAD	2.9094 e5 [m^-1]	3.8924 e4
*v*_*max*_	1.15 10^−17^ [mol/cell s]	1.15 10^−17^ [mol/cell s]
*N*_*v*_	1.12 10^6^ [cell/ml]	1.12 10^6^ [cell/ml]
$\bar{C_{s}}$	168.98 10^−9^ [mol/cl]	168.98 10^−9^ [mol/cl]

In order to complete the mass transport model, we introduced the term *S*, to account for both catabolite production and metabolite consumption in cell metabolism. Given the importance of maintaining cell viability by ensuring sufficient nutrients supply, we focus in particular on metabolite consumption, for which studying transport of oxygen is ideal. Cells are assumed to be confined in the porous scaffold and consumption of nutrients, *S*(*C*_*s*_), is expected to be proportional to their availability, namely *S*(*C*_*s*_) = *S*(*γC*_*s*,*f*_ + (1−*γ*)*C*_*s*,*s*_). Different models can be adopted for this function, either linear or nonlinear. In the former case we set *S*(*C*_*s*_) = *rC*_*s*_, where *r* is a constant parameter determined according to the following balance law:

$$r\cdot \bar{C}=V_{max}\cdot N_{v}$$ (10)

where $\bar{C}$ is a reference concentration for each solute, measured in [mol/ml], *V*_*max*_ is the maximal consumption rate for the considered nutrient and for a specific cell phenotype, quantified in [mol/cell s], and *N*_*v*_ is the average volumetric cell density in the scaffold, measured in [cells/ml]. The main limitation of this model is that it does not guarantee any upper bound for nutrient consumption rate. The more nutrients are available, the more they are metabolized. This approach can be improved using a Michaelis-Menten description of cell metabolism [29], which introduces saturation of the consumption rate, according to the following function:

$$r\left(C_{s}\right)=\frac{V_{max}C_{s}}{K_{m}+C_{s}}$$ (11)

where *K*_*m*_ is the Michaelis-Menten constant, equal to the concentration at which the consumption rate reaches 50% of the maximal value. As a result, the consumption term turns out to be a nonlinear function, namely

$$S\left(C_{s}\right)=r\left(C_{s}\right)C_{s}$$ (12)

We observe that for small nutrient concentrations the linear and the Michaelis-Menten models behave similarly, whereas the latter provides a better estimate of metabolic consumption in case of abundance of nutrients.

### 2.5 Computational solvers

The commercial code ANSYS CFX v.13.0 was used to carry out the fluid dynamic and mass transport simulations. The spatial discretization consists of a cell based finite volume method.

From the computational standpoint, the main challenge of this study consists in solving a fluid-porous interaction problem that involves coupled flow and mass transport. A fully coupled strategy has been adopted, namely all the equations are solved simultaneously through a monolithic linear system that embraces all the degrees of freedom.

More precisely, the Laplace operator in the fluid momentum and oxygen transport equations is approximated by a centered scheme, while the convective terms have been discretized by means of an upwind method. The convective term in the Navier-Stokes equations is linearized by Picard iterations (equivalent to a *fictitious* time stepping method with semi-implicit treatment of $\nabla \cdot \left(\rho \underline{U}\times \underline{U}\right)$) (“ANSYS CFX-Solver Theory Guide”, ANSYS Inc., 2010). The pressure variable in the Navier-Stokes equations is evaluated at the same nodes of the velocity field.

The system is then solved using an algebraic multigrid method exploiting incomplete LU factorization as smoother. Numerical simulations have been performed on parallel CPUs using a quad-socket 12-Core AMD Magny Cours CPU, 128 GB RAM at University of Pittsburgh. Convergence criteria were set to 10^−6^ for the normalized residuals of the global linear system of equations.

To ease the convergence of the algebraic solver, it turned out to be extremely helpful to neglect the contribution of streamline diffusion in the mass transport model, accounting only for the cross-wind component of the diffusion operator. From the modeling standpoint, this approximation is justified since the Péclet number characterizing mass transport in the ducts and in the scaffold of the bioreactor is larger than unity. More precisely, we define the Péclet number as follows

$$Pe=\frac{a\bar{U}}{D}$$ (13)

where *a* is the characteristic length of diffusion, $\bar{U}$ is the characteristic fluid velocity and *D* is the diffusion coefficient of the nutrient in the fluid (water). The Péclet number has been calculated for two sets of parameters, the first one identifying flow and mass transport in the pores of the insert (*a* = 9.77205 e-6 m, $\bar{U}$ = 1.546e-3 m/s, D = 2.9e-9 m^2^/s) and the second one the flow in the chambers that will hold the scaffold (*a* = 5 e-4 [m], $\bar{U}$ = 1.473e-5 [m/s], D = 2.9e-9 [m^2^/s]). For the insert we obtained *Pe* = 2.5, while for the chambers *Pe* = 5.2.

Domain discretization is a crucial phase in the computational model set up to ensure an accurate description of the investigated phenomena as well as reasonable computational time and costs. The geometrical features of the bioreactors span from 8.5 mm (height of the scaffold), to 1 mm (inlet/outlet channel inner diameter), to 0.25 mm (radius of the pores). The final mesh consists of 735658 and 550226 tetrahedral elements for the GelMA and the PLLA case, respectively, with a minimum dimension of the elements of 0.1 mm and a maximum of 0.25 mm. This discretization is suitable for the fluid dynamics model, because, as previously stated, the Reynold’s number results smaller than 0.01, and consequently the boundary layers can be considered fully developed. The fluid dynamics simulations in single array are performed with moderate computational effort (about 7 minutes on CPUs using a quad-socket 12-Core AMD Magny Cours CPU, 128 GB RAM). A numerical test that uses a coarser mesh consisting of 443740 and 242236 elements, respectively, confirms that the results obtained with the finer discretization are insensitive to the mesh size.

## 3 Lumped Parameter Models of HTB

Although in-silico analysis is rightfully considered a cost efficient approach with respect to experimental investigation, section 2.3 illustrates that the development of a computational model of the bioreactor is a challenging task, because of the significant amount of work-hours required to define a detailed CAD model and the considerable computational efforts involved with the definition of a computational mesh and with the solution of the discrete equations.

When using numerical tools in the design or optimization of the bioreactor configuration and working conditions, it is essential to minimize the cost of running simulations for different sets of design parameters. The scientific computing community is well aware of this critical aspect of the approach and has recently made great progress in developing strategies to synthesize surrogate models that replace the brute force simulation approach with much less computational costs. We have mentioned a list of a few examples related to bioengineering [30–36], among many others.

Surrogate or reduced models are based on much simpler mathematical operators than partial differential equations. For steady problems, they may consist of algebraic equations, or ordinary differential equations to capture time dependent phenomena. Such models are often called *lumped parameter models*, because they synthesize into a small number of coefficients the behavior of spatially dependent functions, solutions of partial differential equations, a.k.a. *distributed parameter models*.

The aim of this section is to derive a set of lumped parameter models describing flow and mass transport in the bioreactor fulfilling two objectives:

1. To determine the change of quantitative outputs when the input data are varied, for a fixed single or multi-chamber configuration,
2. To determine the change of quantitative outputs when the number of chambers in the array is varied.

### 3.1 Lumped parameter model for a fixed HTB configuration

We aim to develop an input-output relation between parameters of the model and observed quantities of interest. Because of the linearity of the flow model, motivated by low Reynolds numbers, this relation is a linear operator that can be characterized by a limited number of simulations. The number of required simulations depends on the dimension of the input/output parameter space.

To illustrate the derivation of a lumped parameter model, we consider an example that will be later used for the bioreactor design. In particular, we analyze the flow split at the outlet of the bioreactor chambers for prescribed values of the inlet flow rates.

Let us consider the velocity fields ${\underline{U}}_{i},\;i=1,2$ defined by fixing unit flow rates at each inlet of the bioreactor,

$$\begin{array}{cc} -\nabla \cdot \left(\mu \left(\nabla {\underline{U}}_{i}+{\left(\nabla {\underline{U}}_{i}\right)}^{T}\right)\right)=-\frac{\mu }{K_{perm}}{\underline{U}}_{i}-\nabla p_{i} & \forall x\in {Ω}_{\text{c}\_\text{up}}\cup {Ω}_{\text{c}\_\text{down}}\cup {Ω}_{\text{scaffold}}\\ \nabla \cdot \underline{U_{i}}=0 & \forall x\in {Ω}_{\text{c}\_\text{up}}\cup {Ω}_{\text{c}\_\text{down}}\cup {Ω}_{\text{scaffold}}\\ {\underline{U}}_{i}=1 & \forall x\in \Gamma_{in,i},i=1,2\\ \sigma \left(\underline{U},p\right)\cdot n=0 & \forall x\in \Gamma_{out,i},i=1,2 \end{array}$$ (14)

Since the flow model is linear, the velocity and pressure fields $\underline{U},p$ corresponding to any combination of the inlet flow rates, denoted as *Q*_*in*1_ and *Q*_*in*2_ respectively, can be represented as a linear combination of solutions ${\underline{U}}_{i},p_{i}$

$$\underline{U}=\frac{Q_{in1}}{A_{1}}{\underline{U}}_{1}+\frac{Q_{in2}}{A_{2}}{\underline{U}}_{2};\;p=\frac{Q_{in1}}{A_{1}}p_{1}+\frac{Q_{in2}}{A_{2}}p_{2}$$ (15)

Since we are interested in the quantification of the outflow rates, we calculate

$$\begin{array}{c} Q_{out,i}=\int_{\Gamma_{out,i}}\underline{U}\cdot \underline{n}dx=Q_{in,1}\overset{m_{i,1}}{\overset{︷}{\int_{\Gamma_{out,i}}{\underline{U}}_{1}\cdot \underline{n}dx}}+Q_{in,2}\overset{m_{i,2}}{\overset{︷}{\int_{\Gamma_{out,i}}{\underline{U}}_{2}\cdot \underline{n}dx}}=\\ =Q_{in,1}\cdot m_{i,1}+Q_{in,2}\cdot m_{i,2} \end{array}$$ (16)

As a result, we have identified the following input-output algebraic relation between inlet and outlet flow rates

$$\left|\begin{array}{c} Q_{out,1}\\ Q_{out,2} \end{array}\right|=\left|\begin{array}{cc} m_{1,1} & m_{1,2}\\ m_{2,1} & m_{2,2} \end{array}\right|\cdot \left|\begin{array}{c} Q_{in,1}\\ Q_{in,2} \end{array}\right|\;i.e.\;\left|\begin{array}{c} Q_{out,1}\\ Q_{out,2} \end{array}\right|=M\;\left|\begin{array}{c} Q_{in,1}\\ Q_{in,2} \end{array}\right|\;M=\left|\begin{array}{cc} m_{1,1} & m_{1,2}\\ m_{2,1} & m_{2,2} \end{array}\right|$$ (17)

that represents the lumped parameter model we were looking for. We note that the operator (matrix) *M* depends on the bioreactor geometric design.

This approach can be extended to the mass transport problem, provided that the model adopted for consumption of nutrients is linear, namely *S*(*C*_*s*_) = *rC*_*s*_. In this case, we denote with *d*_*i*_ the solution of Eq 5 obtained setting ${\bar{C}}_{i}=C_{in,i}=1$ and ${\bar{C}}_{j\neq i}=C_{in,i\neq j}=0$. Then, any solution *C*_*f*_ of the mass transport problem can be expressed as

$$C_{f}=C_{in,1}d_{1}+C_{in,2}d_{2}$$ (18)

Let *C*_*out*,1_,*C*_*out*,2_ be the nutrient concentration on the upper and lower outlets respectively and for simplicity of notation let us define

$$d_{i,1}={d_{i}|}_{\Gamma \text{out},\text{up}},d_{i,2}={d_{i}|}_{\Gamma \text{out},\text{down}}$$ (19)

Then, because of the linearity of the mass transport model we obtain

$$C_{out,i}=C_{in,1}\cdot d_{1,1}+C_{in,2}\cdot d_{2,1}$$ (20)

that can be translated in the following vector form,

$$\underline{C_{out}}=D\cdot \underline{C_{in}};\;\underline{C_{out}}=\left|\begin{array}{c} C_{out,1}\\ C_{out,2} \end{array}\right|,\;\underline{C_{in}}=\left|\begin{array}{c} C_{in,1}\\ C_{in,2} \end{array}\right|,\;D=\left|\begin{array}{cc} d_{1,1} & d_{1,2}\\ d_{2,1} & d_{2,2} \end{array}\right|$$ (21)

### 3.2 Lumped parameter model for variable bioreactor configurations

Here we focus on the problem of determining a lumped parameter model for a sequence of bioreactor units, when the solution for 1-unit is known. From the methodological standpoint, this problem is more challenging than the one of characterizing the lumped parameter model for one bioreactor unit, because partial differential equations are not linear with respect to the configuration of the domain. In other words, the solution of an *n*-unit bioreactor is not the superposition of *n* solutions of a single unit configuration.

**Fig 4:**
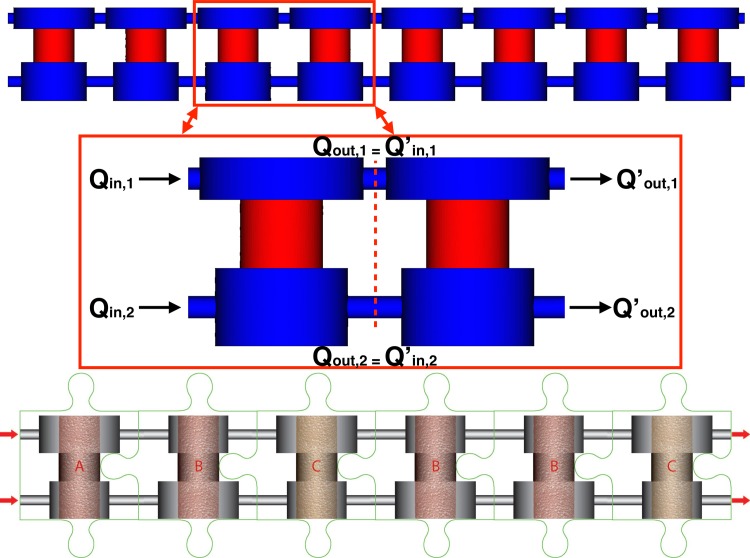
Distribute vs lumped parameter models Top: A 8-unit bioreactor configuration, showing details of a 2-unit example used for the development of the lumped parameter model (top panels). Bottom: A sketch of a multi-unit bioreactor configuration with heterogeneous unit design in a generic sequence of units, where different unit designs are denoted with letters A, B, C.

Another strategy for determining a lumped parameter model of a multi-unit configuration emerges observing that units are combined in sequence (see Fig 4). Consequently, we conjecture that the behavior of the *n*-unit bioreactor is the *composition* of *n-unit* models. As an example, for a sequence of two units we posit that the input/output relation for flow rates is

$$\left|\begin{array}{c} Q\prime_{out_{2}}\\ Q\prime_{out_{1}} \end{array}\right|=\tilde{M}\left|\begin{array}{c} Q_{in_{2}}\\ Q_{in_{1}} \end{array}\right|;\;\left|\begin{array}{c} {Q^{\prime }}_{out_{2}}\\ {Q^{\prime }}_{out_{1}} \end{array}\right|={\tilde{M}}_{1}\left|\begin{array}{c} {Q^{\prime }}_{in_{2}}\\ {Q^{\prime }}_{in_{1}} \end{array}\right|;\;\left|\begin{array}{c} {Q^{\prime }}_{in_{2}}\\ {Q^{\prime }}_{in_{1}} \end{array}\right|={\tilde{M}}_{2}\left|\begin{array}{c} Q_{in_{2}}\\ Q_{in_{1}} \end{array}\right|$$ (22)

Owing to the similar design of the upper and lower chambers, the resistance to flow of the fluid entering from the upper and lower inlets is comparable. As a result, the following property is valid at any junction between two adjacent bioreactor units,

$${\sigma \left(\underline{U},p\right)\cdot n|}_{\Gamma_{1}}={\sigma \left(\underline{U},p\right)\cdot n|}_{\Gamma_{2}}$$ (23)

It shows that equal normal stresses are applied at the intermediate section of a 2-unit bioreactor. Since these are the boundary conditions applied at the outlet of our model for an individual unit it means that any unit in a row functions as an individual one. As a result, we conclude that

$${\tilde{M}}_{1}≅M;\;{\tilde{M}}_{2}=M$$ (24)

and consequently

$$\left|\begin{array}{c} {Q^{\prime }}_{out_{2}}\\ {Q^{\prime }}_{out_{1}} \end{array}\right|=M\cdot \left|\begin{array}{c} {Q^{\prime }}_{in_{2}}\\ {Q^{\prime }}_{in_{1}} \end{array}\right|=M\cdot M\left|\begin{array}{c} Q_{in_{2}}\\ Q_{in_{1}} \end{array}\right|=M^{2}\left|\begin{array}{c} Q_{in_{2}}\\ Q_{in_{1}} \end{array}\right|$$ (25)

This example can be easily generalized to the case of a row of *n*-units. More precisely, we infer that the lumped parameter model for an *n*-unit bioreactor, denoted by *M*_*n*_ is the *multiplicative composition of n single unit models*, namely

$$M_{n}=M^{n}$$ (26)

where the latter expression denotes the *n*-th power of the operator *M*.

This approach can be applied to flow (as illustrated above) as well as to mass transport. In this way, the lumped parameter models *M*,*D*, derived in section 3.1 for single unit configurations, can be extended to multi-unit configurations made of units combined in a row. Using direct numerical simulations of multi-cell configurations, we will demonstrate in the next sections the good accuracy of these reduced models.

We finally observe that the model composition rule is also applicable in the case of combination of different unit designs (schematized in Fig 4 with letters A, B, C). In particular, the input/output relation (*Y* = *M* ∙ *X*) for a row of 3-units of generic type *A*, *B*, *C* of which we know the individual lumped parameter models, *M*_*A*_,*M*_*B*_,*M*_*C*_ respectively, is given by *M* = *M*_*A*_ ∙ *M*_*B*_ ∙ *M*_*C*_. Following the ambitious vision of building a *human-on-chip* model, any pattern of bioreactors organized in a row can be characterized using this approach, provided that the properties of each individual unit are known.

## 4 Numerical Simulations

### 4.1 Numerical simulation of flow

In this study, simulations of flow are performed to compare flow patterns in the GelMA and PLLA scaffold when inlet flow rates are varied. More precisely, the following different flow pairs were simulated: (a) 1 and 1, (b) 1 and 2 and (c) 10 and 10 ml/day for the upper and lower inlet, respectively.

**Fig 5:**
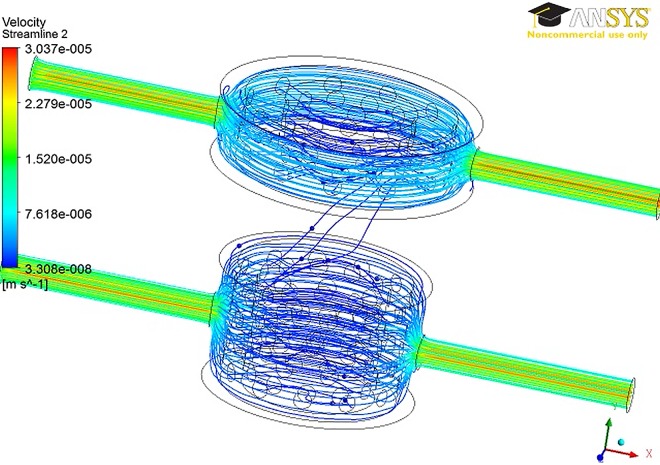
Streamlines in the 1- unit model with the GelMA scaffold.

We observe that for all the configurations, the fluid is driven by the pressure gradient to move toward the upper chamber (Fig 5). The flow split obtained by applying the different flow pairs are reported in Tables 3 and 4 for the GelMA and PLLA case, respectively. The comparison of the outlet flow rates for the two scaffolds highlighted opposite outcomes in terms of flow mixing. Indeed, while not significant flow mixing was found for the GelMA scaffold, a significant mixing occurs in the PLLA case. As expected, the maximum mixing (that is 42.9%) occurs with different input fluid flow rates (1 and 2 ml/day at the upper and lower inlet, respectively).

**Table 3:** Results obtained by simulating different flow split in the one unit model with the GelMA and PLLA scaffold.

	Inlet		GelMA		PLLA	
	Q_in,top_ [Kg/s]	Q_in,down_ [Kg/s]	Q_out,top_ [Kg/s]	Q_out,down_ [Kg/s]	Q_out,top_ [Kg/s]	Q_out,down_ [Kg/s]
a)	1	1	1	1	1.032	0.968
b)	1	2	1	2	1.429	1.571
c)	10	10	10	10	10.32	9.68

**Table 4:** Percentage of oxygen consumption for the GelMA and PLLA scaffold.

	GelMA	PLLA
Upper chamber	0.93%	4.9%
Lower Chamber	1.8%	8.14%

For the sake of brevity, the results of the 4-units array are not reported since they are the qualitatively equivalent to the single unit configuration.

### 4.2 Numerical simulation of transport

Simulations of oxygen transport were performed to compare mass transfer in the GelMA and PLLA scaffolds.

**Fig 6:**
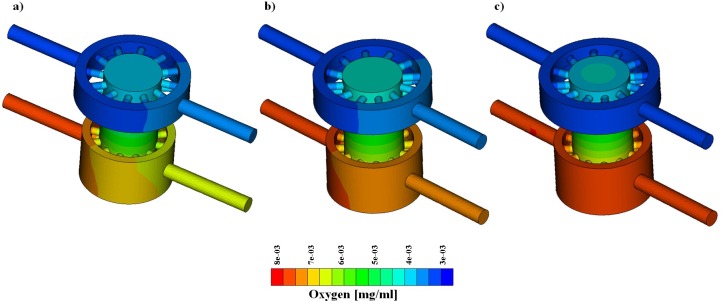
Oxygen concentration with GelMA scaffold. From left to right, flow pair of 1–1 [ml/day], 1–2 [ml/day], 10–10 [ml/day].

**Fig 7:**
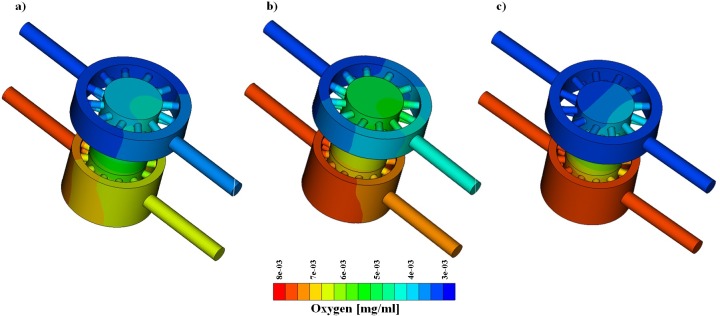
Oxygen concentration with PLLA scaffold. From left to right, flow pair of 1–1 [ml/day], 1–2 [ml/day], 10–10 [ml/day].

Concentrations equal to 3.15 and 7.2 μg/l were applied at the upper and lower inlet, respectively. As in the previous case, the following flow pairs were simulated: (a) 1 and 1, (b) 1 and 2 and (c) 10 and 10 ml/day at the upper and lower inlet. Two configurations of the bioreactor were considered, namely 1-unit and a 4-unit array. The results of 1-unit model are reported in Fig 6 and Fig 7 for the GelMA and PLLA scaffold, respectively. The analysis of the mass transport simulations obtained for the GelMA and the PLLA scaffolds allows us to draw general considerations, which are valid for both single and 4-unit arrays.

Firstly, as explained in section 2.3.2, we see that axial advection is dominant with respect to the cross-wind diffusion. Therefore, the higher the flow rates and fluid velocity, the more the inlet and outlet oxygen concentrations look similar due to a reduced oxygen drop (Fig 6B and Fig 7B). However, the diffusion of oxygen from the lower chamber to the upper one is not negligible, because different inlet concentrations promote the formation of concentration gradients that trigger transport.

For both the GelMA and PLLA cases, the oxygen concentration in the top region of the scaffold is higher in the case of low flow rate, (a, inlet flow equal to 1 ml/day) than in the case of high flow rate (c, inlet flow equal to 10 ml/day). Concerning case (b), the mix of the two chambers’ flow is greater and a contribution of convective transport is added to the diffusive flux from the bottom towards the top of the bioreactor chamber. For this reason, the oxygen concentration in the top region of the scaffold is greater in case (b) than in cases (a) and (c).

Finally, the simulations suggest that the scaffold porosity and permeability play a relevant role on mass transport. Indeed, while the GelMA is permeable to oxygen, the PLLA is not. This implies that the aforementioned phenomena are more evident with a polymeric scaffold impervious to mass transport through the solid phase, such as PLLA.

### 4.3 Oxygen consumption

The simulations of oxygen consumption were performed for the two different scaffolds (GelMA and PLLA) for an array of 4-units, in order to study the depletion of nutrients in the culture medium. The flow split is the one of case (a) (1 and 1 ml/day) and the inlets concentrations are equal to 3.15 and 7.2 μg/l at the upper and lower inlet, which correspond to the normoxic levels of the different types of tissue grown in the upper and lower chambers.

**Fig 8:**
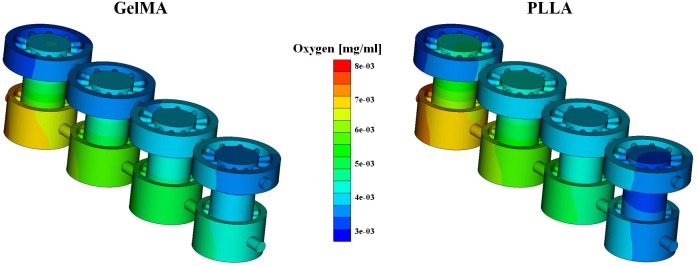
Oxygen concentration in the 4 cells array. GelMA (left) and PLLA (right) scaffolds are used when with active consumption rate.

Since we consider a 4-unit array, we observe that diffusion develops more easily along the bioreactor axis (longer fluid path with respect to the 1-unit case) and as a consequence, the oxygen concentration tends to become more uniform. More precisely, enhanced diffusion combined with different inlet concentrations causes a decrease of the oxygen level in the lower chamber and an increase in the top one. This trend is heightened by cellular oxygen consumption, which further leads to a diminishing of the oxygen concentration in the lower chamber (Fig 8).

The two types of scaffold show the same trend of oxygen consumption, but the computations highlighted different percentage of consumed oxygen. Indeed, a higher percentage of oxygen consumption was found for the PLLA scaffolds with respect to GelMA. This effect is likely a result of the different cell density used for the two cases. In fact, cell density is assumed to be equal to 1 x 10^6^ cells/ml for GelMA and to 2.12304019 x 10^6^ cells/ml in the case of PLLA.

### 4.4 Comparison of distributed and lumped parameter models

In this section, the results of the lumped and the distributed parameter models are presented and compared in terms of fluid dynamics and mass transport. The fluid dynamics results for 1-unit and 4-units array are first presented, then, the mass transport results of both configurations are studied. For the sake of brevity, we present only the results obtained by simulating the GelMA scaffold.

#### 4.4.1 Fluid dynamics

Two computational fluid dynamics simulations were performed for the single unit configuration to determine the lumped parameter model (LPM). In particular, two inlet flow pairs are applied as reported in Table 5. The resulting LPM matrix M is:

$$M=\left[\begin{array}{cc} 1 & 6.820\;e-8\\ 6.188\;e-8 & 0.9999 \end{array}\right]$$

**Table 5:** Simulation settings to identify the fluid dynamics characteristics of one-unit bioreactor.

	Q_in,top_ [Kg/s]	Q_in,down_ [Kg/s]	Q_out,top_ [Kg/s]	Q_out,down_ [Kg/s]
#1	1e-08	0	1e-08	6.19e-16
#2	0	1e-08	6.82e-16	9.99e-9

Then, the results of the 1-unit and 4-unit LPMs are compared to those of the distributed parameter model, see Tables 6 and 7, and in two test cases the error was lower than 1%.

**Table 6:** Comparison of the 1-unit fluid dynamics results provided by the distributed (distr) and the lumped (lump) parameter models.

	Q_in,top_ [Kg/s]	Q_in,down_ [Kg/s]	Q_out,top,distr_ [Kg/s]	Q_out,down,distr_ [Kg/s]	Q_out,top,lump_ [Kg/s]	Q_out,down,lump_ [Kg/s]
#1	1.157e-08	1.157e-08	1.157e-08	1.157e-08	1.157e-08	1.569e-08
#2	1.157e-08	2.314e-08	1.157e-08	2.314e-08	1.157e-08	2.313e-08

**Table 7:** Comparison of the 4-unit array fluid dynamics results provided by the distributed (distr) and the lumped (lump) parameter models.

	Q_in,top_ [Kg/s]	Q_in,down_ [Kg/s]	Q_out,top, distr_ [Kg/s]	Q_out,down, distr_ [Kg/s]	Q_out,top, lump_ [Kg/s]	Q_out,down, lump_ [Kg/s]
#1	1.157e-08	1.157e-08	1.157e-08	1.157e-08	1.157e-08	1.569e-08
#2	1.157e-08	2.314e-08	1.157e-08	2.314e-08	1.157e-08	2.313e-08

#### 4.4.2 Mass transport

For the LPM model of mass transport we have adopted the parameters of Table 2 and inlet concentrations summarized in Table 8.

**Table 8:** Simulation settings to identify the mass transport input-output characteristics of one-unit bioreactor.

	[O_2_]_in,top_ [mg/ml]	[O_2_]_in,down_ [mg/ml]	[O_2_]_out,top_ [mg/ml]	[O_2_]_out,down_ [mg/ml]
#1	1e-03	0	8.514e-04	1.486e-04
#2	0	1e-03	1.486e-04	8.514e-04

To start with, we analyze the mass transport model without cell metabolism, that is the case *S*(*C*_*s*_) = 0 in Eq 5. The LPM model for the corresponding mass transport simulations is the following matrix:

$$D=\left[\begin{array}{cc} 0.8481 & 0.1519\\ 0.1519 & 0.8481 \end{array}\right]$$

The results of the 1-unit LPM are compared with those of the distributed parameter model in two simulations with different inlets concentrations, reported in Table 9, whose values are set according to ongoing experimental tests. The results from the LPM model differ from those of the distributed parameters model by less than the 1%.

**Table 9:** Comparison of the one-unit oxygen concentration results provided by the distributed (distr) and the lumped (lump) parameter models.

	[O_2_]_in,top_ [mg/ml]	[O_2_]_in,down_ [mg/ml]	[O_2_]_out,top,distr_ [mg/ml]	[O_2_]_out,down,distr_ [mg/ml]	[O_2_]_out,top,lump_ [mg/ml]	[O_2_]_out,down,lump_ [mg/ml]
#1	3.15e-03	7.2e-03	3.765e-03	6.585e-03	3.765e-03	6.585e-03
#2	2e-03	4e-03	2.304e-03	3.696e-03	2.304e-03	3.696e-03

We also calculate the LPM model for mass transport with active cell metabolism. For the linear model, *S*(*C*_*s*_) = *rC*_*s*_, the LPM matrix for 1-unit is the following

$$D_{l}=\left[\begin{array}{cc} 0.6550 & 0.137\\ 0.137 & 0.605 \end{array}\right]$$

while for the Michaelis-Menten case, namely Eqs 11 and 12, the LPM model becomes

$$D_{mm}=\left[\begin{array}{cc} 0.8377 & 0.1347\\ 0.1327 & 0.8147 \end{array}\right]$$

**Fig 9:**
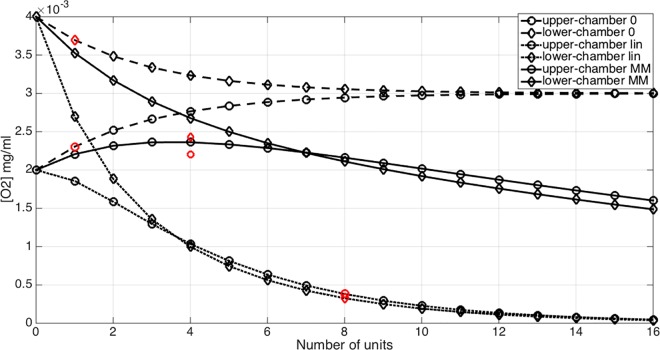
Variation of the outlet concentration of oxygen with respect to the number of units (unit #0 denotes the inlet value) for the mass transport model without cell metabolism (dashed line), with linear consumption rate (dotted line) and with Michaelis-Menten consumption model (solid line). Data calculated using the full 3D model are reported in red.

The inspection of the matrices *D*,*D*_*l*_,*D*_*mm*_ informs about the characteristics of the different consumption models compared here. We observe that the diagonal entries of *D*_*l*_ are the smallest, confirming that the linear model is the one with the highest oxygen consumption rate. The extra-diagonal coefficients correspond to the oxygen exchange between the upper and lower chambers. Their magnitude is similar in all cases, because they depend on the diffusion parameters solely. For the linear case, the theory at the basis of the LPM derivation is satisfied, while it does not rigorously hold true for the Michaelis-Menten model, because the mass transport equation becomes nonlinear. Once again, numerical simulations based on the full model applied to the 8-unit array confirm that the LPM model with linear consumption rate, namely *D*_*l*_, predicts outlet concentrations with less than 1% error. The corresponding results are reported in Table 10 and visualized in Fig 9. In Table 11 we report the error of the LPM based on the Michaelis-Menten nonlinear consumption rate. Despite the nonlinear nature of the problem, in conflict with the principles at the basis of the LPM derivation, the LPM model is fairly accurate in predicting the concentration split and decay at the outlet also with a Michaelis-Menten consumption rate, with a maximum error of about 10% for an array of 4-units, located on the bottom outlet of the bioreactor.

**Table 10:** Comparison of the 8-unit array oxygen concentration results provided by the distributed (distr) and the lumped (lump) parameter models with linear consumption rate.

	[O_2_]_in,top_ [mg/ml]	[O_2_]_in,down_ [mg/ml]	[O_2_]_out,top, distr_ [mg/ml]		[O_2_]_out,down,distr_ [mg/ml]	[O_2_]_out,top,lump_ [mg/ml]	[O_2_]_out,down,lump_ [mg/ml]
#1	3.15e-03	7.2e-03	6.555 e-4	5.628 e-4		6.545 e-4	5.613 e-4
#2	2e-03	4e-03	3.826 e-4	3.274 e-4		3.821 e-4	3.265 e-4

**Table 11:** Comparison of the 4-unit array oxygen concentration results provided by the distributed (distr) and the lumped (lump) parameter models with Michaelis-Menten consumption rate.

	[O_2_]_in,top_ [mg/ml]	[O_2_]_in,down_ [mg/ml]	[O_2_]_out,top,distr_ [mg/ml]	[O_2_]_out,down,distr_ [mg/ml]	[O_2_]_out,top,lump_ [mg/ml]	[O_2_]_out,down,lump_ [mg/ml]
#1	3.15e-03	7.2e-03	3.754e-03	4.295e-03	3.971e-03	4.678e-03
#2	2e-03	4e-03	2.202e-03	2.43e-03	2.3482e-03	2.677e-03

The LPM model for mass transport is particularly interesting because it allows us to estimate the decay of nutrient concentrations due to cell metabolism along an arbitrarily long array of units, using the formula $\underline{C_{out}}\left(n\right)=D^{n}\cdot \underline{C_{in}}$. Considering for example the inlet concentrations of Table 9, test case #2 for $\underline{C_{in}},$ we estimate the outlet concentration decay for the transport model without oxygen consumption. The same calculation is then repeated for the linear and the Michaelis-Menten models for cell metabolism and the results are compared in Fig 9, where also the outlet concentrations determined using the fully 3D simulations are shown for a qualitative visualization of the LPM error.

## 5 Discussion

From the engineering standpoint, our study shed lights on important aspects of the bioreactor behavior. We observe that the flow is dominated by viscous effects and by pressure gradients, while inertial effects are negligible. Differences in inlet velocities between upper and lower chamber generate a vertical pressure gradient inside the bioreactor chambers, which promotes mixing of nutrient fluid flowing through the osteochondral construct. Furthermore, we have observed that the magnitude of vertical pressure gradients depends highly on the permeability of the scaffold. Between the two materials tested here, it appears that the most permeable one favors the mixing of fluid among the upper and lower chambers.

Concerning mass transfer, our simulations suggest that it is dominated by convection. Diffusion effects are however non-negligible, but their (relative) intensity varies according to the inlet flow rate and the scaffold properties. More precisely, Fig 6 and Fig 7 show that high flow rates decrease the transport of biochemical species between the two chambers. From the analysis of these plots we also observe that the concentration in the bioreactor top chamber is greater than the one at the upper outlet. This means that the exchange between the chamber and the supplying channels is not sufficient to remove all the chemical species that accumulate in this region, because of combined diffusion and convection. This effect is observable for both types of scaffold, but is more evident for GelMA, suggesting that this type of material hinders flow and mass transport more than PLLA does. When nutrient (or oxygen) consumption is switched on in the simulation, concentration gradients are quickly smoothed out when traveling along multiple bioreactor units. At the same time, concentration levels significantly decrease. The computational model thus serves as a valuable tool to estimate whether the final units of the row receive enough nutrients, as illustrated in the example presented above.

Finally, we have developed a surrogate, inexpensive approach to characterize the output of the bioreactor without the burden of running many computer simulations. It consists of a lumped parameter model, derived exploiting the linearity of the full model. The LPM has proven to be very accurate in capturing the effect of sequentially combining multiple units. A natural application of this model is studying the concentration decay along a sequence of bioreactor units. For example, Fig 9 shows the concentration decay at the bioreactor outlets when the number of units is varied from 1 to 16. Three sets of curves outline the behavior of different cell metabolism models. When cell metabolism is switched off (dashed lines), the upper and lower concentrations equilibrate very quickly, confirming that diffusion effects of oxygen between the two chambers are non negligible. We recall that large oxygen diffusion and transport between the upper and lower chambers is not necessarily desirable, when different types of tissue are grown. Indeed, in our case, cartilage natural environment should be hypoxic, while bone better develops in normoxic conditions. For constant consumption rate, the concentration decay is the largest. As a consequence after 16 bioreactor units, almost all the nutrient concentration has been consumed. The Michaelis-Menten metabolic model is the most realistic of the three options, because it accounts for a saturation effect that limits the consumption rate. According to our preliminary data on cell viability in the bioreactor, obtained by Live/Dead assays (data not shown), the oxygenation computed after 16-units appears to be still at a sufficient level.

The computational approach proposed here is subject to some limitations. One is the approximation of the fluid dynamic and mass transport through steady model. A key challenge in the engineering of three-dimensional tissue is maintenance of cell viability when the volumetric cell density increases. In this study, we assumed a constant cell density equal to the initial culture conditions that occur after distributing cells homogenously throughout the volume of the scaffolds. However, variations in cell density with time could be easily incorporated in both our models, to predict oxygen drops in long-term culture. Secondly, as literature data are lacking, we assumed the GelMA properties (i.e., porosity and permeability) equal to those of native cartilage. Experimental tests will be performed in future work to assess these properties. Finally, we have not accounted for transport along capillaries. This could be acceptable for many engineered constructs that are approximation of native tissues, frequently obtained from single cell types, e.g., mesenchymal stem cells, within a hydrogel or a porous scaffold. If the HTB were to be used with native tissues, we expect our approach to hold true with the necessary adjustments to account for the different tissues types. The avascular components of cartilage would be modeled adjusting the parameters we currently used for GelMA, whereas the for the vascularized bone, the more porous structure we described for the PLLA scaffold could offer a good starting model to approximate the cavities and capillaries present in subchondral bone.

Another improvement of our study would be to validate the oxygen concentration drops predicted by our models with actual measurements performed when the bioreactor is operated with cell-seeded constructs. This validation would be technically challenging, only feasible using oxygen sensors incorporated in the perfusion circuit, at the inlet and outlets of each bioreactor unit or even inserted directly in the chambers, in direct contact with the living cells [37]. Detecting larger molecules, even at low concentrations provides a more simple and reliable quantification. On this basis, extensive validation of the ability of our models to predict the flow-dynamics and mass transport in the bioreactor will be the subject of future work.

## 6 Conclusions and Perspectives

From the methodological standpoint, we have overcome the challenge of developing a complex multi-physics model of the bioreactor. We have also succeeded in implementing the model into a commercial computational platform, showing the significant potential of computational tools on biomedical research, including analytical cases integrating quantitative biology and translational medicine. Future developments of this study consist of experimental validation of the models and their application to explore different bioreactor configurations. Such findings will allow optimization of the model by incorporating the multi-faceted factors that affect its behavior and functionality.
